# The Sponge Tent—Its Preparation and Use

**Published:** 1868-12

**Authors:** 


### The Sponge Tent---Its Preparation and Use.

Dr. George Syng Bryant, of Louisville, Ky., (late of this city,) says, that for the past eighteen months he has prepared an *antiseptic* sponge tent, as follows: He selects moderately coarse, elastic sponge, which, being well cleansed, and while wet, cut into the shape and size required, is then saturated with thick gum mucilage, prepared by using ten or twelve grains of crystalized carbolic acid to the ounce, and wrapped on an awl, with a strong, well-twisted cord. The tent should be fusiform, and wrapped from the small end, taking care that the layers of the cord are carried around in close proximity and with perfect regularity; and by retaining the "screw threads" thus formed, and turning it as an ordinary screw during insertion, the tent can be more easily introduced, and will not slip out as a smooth one is apt to do. To facilitate introduction, the tent, more particularly at the small extremity, should always be slightly soaped, when, firmly grasped by a pair of small, straight forceps with an attachment for holding the blades fast, it should be inserted as directed, so as not to project more than one-eighth or one-fourth of an inch out of the os, the uterus being held by a volsella or hook. Among the numerous uterine diseases and derangements which are most frequently benefited by its use, he mentions the following: Granular erosions of the os and cervix uteri, which, when thus treated, seldom fail to disappear in a short time, and the mucous membrane becomes smooth and natural.

Fungoid granulations, often a source of great annoyance to the surgeon, and distress to the patient, soon disappear under proper use of the tent.

In fibrinous infiltration of the os and cervix, either alone or in complication with many diseased states of the uterus, as erosion, fungoid granulations, sub-involution, ulceration, elongated neck, constrictions, and flexions, with or without retroversion or antiversion, not only will the infiltration disappear under the use of the sponge tent, but a'so, the complicating disease.

In pathological hypertrophy, either with or without chronic inflammation, the sponge tent acts with peculiar benefit, exciting the absorbents to take up all superfluous deposits or tissue. Intrauterine fibroid and polypoid tumors, when of small size, will sometimes be completely destroyed by the sponge tent in a few days.

If the fibroid be small and intra-mural, it may be reduced in size, and possibly, in some instances, be removed completely, by the long continued use of the sponge tent, resting the patient three or four days in every week.

Polypoids, when attached to the canal of the cervix, if of small size, yield readily to the pressure of the sponge tent.

In the treatment of uterine diseases, the bowels, he says, must be kept in a soluble state, much depending on the condition in the treatment of most uterine affections. —*Humboldt Med. Archives.*

